# Efficacy of ultrasound-guided recto-intercostal fascial plane block for postoperative analgesia in laparoscopic cholecystectomy: a randomized controlled trial

**DOI:** 10.1007/s00464-026-12787-5

**Published:** 2026-04-17

**Authors:** Hande Gungor Danisan, Burak Omur, Ayse Ince, Birzat Emre Golboyu, Yasar Ozdenkaya, Tumay Uludag Yanaral, Cem Gezen, Bahadır Ciftci

**Affiliations:** 1 Department of Anesthesiology and Reanimation, Istanbul Medipol University, Istanbul, Turkey; 2 Department of Anesthesiology and Reanimation, Katip Çelebi University, Izmir, Turkey; 3 Department of General Surgery, Istanbul Medipol University, Istanbul, Turkey; 4 Department of Anatomy, Istanbul Medipol University, Istanbul, Turkey; 5 Faculty of Medicine Anesthesiology and Reanimation Department, Istanbul Medipol University, 34214 Istanbul, Turkey

**Keywords:** Analgesia, Laparoscopic cholecystectomy, Nerve block, Postoperative pain, Rectus muscle of abdomen, Ultrasonography

## Abstract

**Background:**

The recto-intercostal fascial plane block (RIFPB) is a novel interfascial plane block technique that targets the anterior and lateral cutaneous branches of the T6–T9 thoracoabdominal nerves. The purpose of this study was to assess RIFPB’s analgesic effectiveness in managing postoperative pain in patients having laparoscopic cholecystectomy (LC).

**Methods:**

Patients scheduled for elective LC under general anesthesia who were between the ages of 18 and 65 and had ASA physical status I–II were included. Patients were divided into two groups at random: the control group (n = 38) and the RIFPB group (n = 39). While the control group received normal multimodal postoperative analgesia, the RIFPB group underwent bilateral RIFPB. The primary outcome was the need for rescue analgesia. Secondary outcomes included postoperative pain scores, and the incidence of adverse effects.

**Results:**

When comparing the RIFPB group to the control group, the incidence of rescue analgesia was considerably lower (17.9 vs. 55.3%, p = 0.001), and total rescue analgesic (tramadol) consumption was reduced [0 (0–0) mg vs. 40 (0–50) mg, p = 0.001]. At 1, 3, 6, and 12 postoperative hours, the RIFPB group had significantly decreased pain scores (p < 0.05). The incidence of postoperative nausea, vomiting and itching was lower in the RIFPB group.

**Conclusions:**

RIFPB provided effective postoperative pain control and reduced rescue analgesic requirements compared with standard analgesic management.

Research registration number: NCT06768593.

Laparoscopic cholecystectomy (LC) is the standard surgical treatment for gallbladder diseases and is associated with lower morbidity, shorter hospital stay, and faster recovery compared with open surgery. However, despite being minimally invasive, postoperative pain remains a clinically significant problem, negatively affecting early mobilization, oral intake, and patient satisfaction. Inadequately controlled postoperative pain may lead to adverse outcomes such as respiratory depression, pulmonary complications, and prolonged hospitalization [1, 2]. Postoperative pain following laparoscopic cholecystectomy is multifactorial, comprising somatic pain originating from trocar insertion sites, visceral pain related to peritoneal distension and CO_2_ insufflation, and shoulder pain secondary to diaphragmatic and phrenic nerve irritation. Therefore, effective postoperative analgesia requires multimodal approaches targeting both somatic and visceral pain components to optimize analgesia, reduce opioid consumption, and minimize opioid-related adverse effects such as nausea and vomiting [3, 4].

Current postoperative analgesic strategies include opioids, non-opioid analgesics (NSAIDs and paracetamol), local anesthetic infiltration, and various regional anesthesia techniques. Due to the undesirable effects of opioids, such as nausea, vomiting, sedation, and respiratory depression, interest in regional anesthesia techniques has increased. Among these, ultrasound-guided fascial plane blocks have gained popularity owing to their ease of application and low complication rates [5–7]. In upper abdominal and laparoscopic procedures, a number of fascial plane blocks have been reported and are frequently utilized to provide analgesia [8–11].

The recto-intercostal fascial plane block (RIFPB) is a novel interfascial block method, in which local anesthetic is injected into the fascial plane between the rectus abdominis muscle and the 6th–7th costal cartilages, thereby blocking the anterior and lateral cutaneous branches of the T6–T9 thoracoabdominal nerves [12]. Following its initial description, the clinical applications of RIFPB have rapidly expanded, and several case reports have demonstrated its successful use in different clinical settings [13–16].

The aim of this study was to evaluate the effectiveness of ultrasound-guided RIFPB for postoperative pain control following LC by comparing it with conventional port-site infiltration, which represents routine analgesic practice. We hypothesized that RIFPB would provide more effective postoperative analgesia than the control group.

## Methods

### Study design

The Institutional Research and Ethics Board approved this prospective, randomized, controlled clinical trial (Decision No: 1192, Date: 28 November 2024). Following ethical approval, the study was registered at ClinicalTrials.gov (NCT06768593) prospectively, and patient recruitment subsequently began in January 2025. The study was conducted in accordance with the Declaration of Helsinki and continued until October 2025 at Medipol Mega University Hospital.

**Fig. 1:**
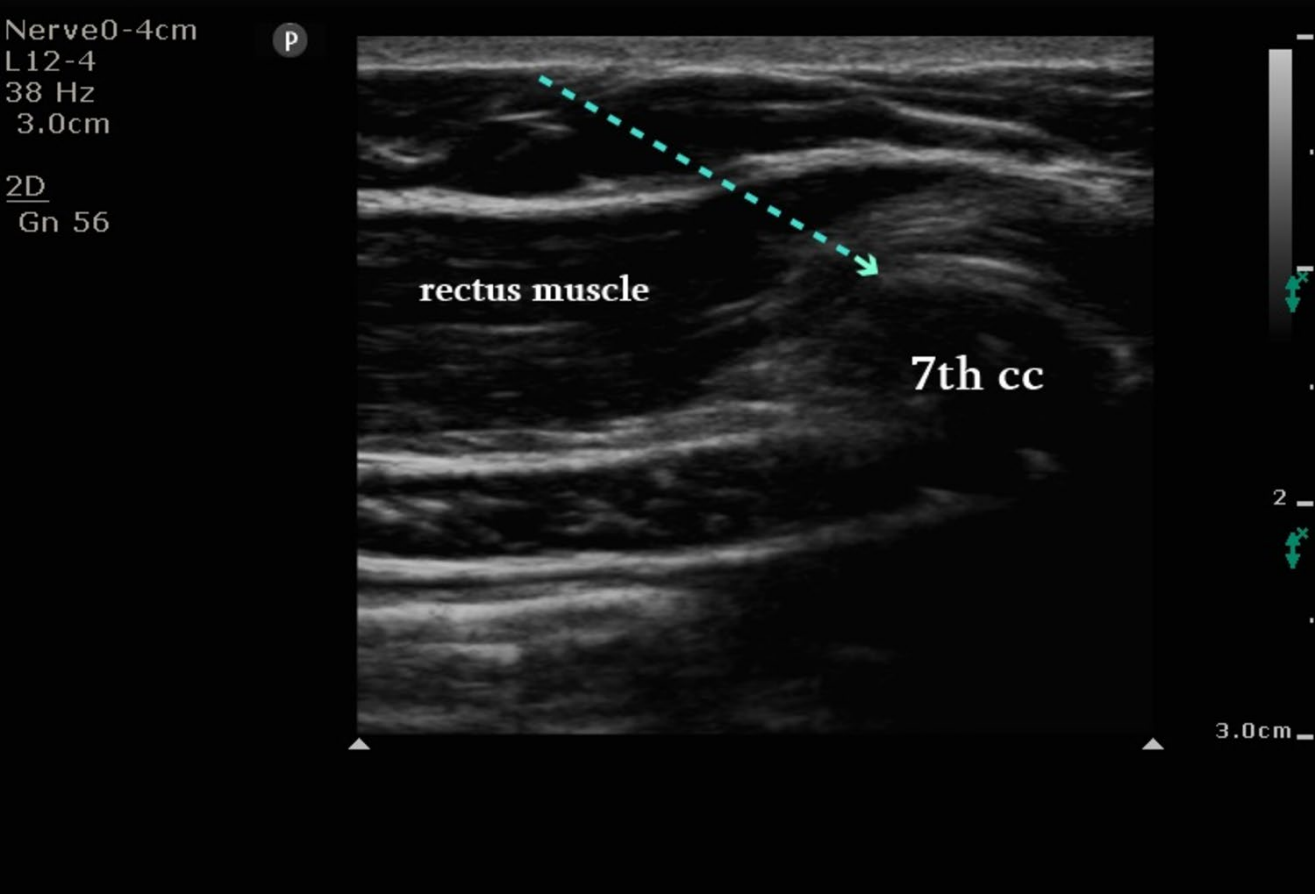
Sonographic visualization of RIFPB. The arrow indicates the needle trajectory, cc costal cartilage

A total of 86 individuals with ASA physical status I–II who had planned for elective laparoscopic cholecystectomy and ranged in age from 18 to 65 were evaluated for participation. Eight patients were excluded because they did not meet the inclusion criteria, and 78 patients were enrolled in the study; RIFPB group: 39 patients, and control group: 39 patients. In the control group, one patient was excluded from the analysis due to intraoperative conversion from laparoscopic to open surgery (Fig. 1). Patients with known allergy to local anesthetics or opioids, coagulopathy or anticoagulant use, infection or skin lesions at the block site, alcohol or substance abuse, pregnancy, or those who declined participation were excluded.

### Grouping and randomization

Patients were divided into two groups at random prior to entering the operating room: the RIFPB group (n = 39) and the control group (n = 39). The Research Randomizer software was used to perform randomization. Every patient was given a distinct random identification number and a randomization table was created. Every patient’s group assignment was concealed. Because the block was performed under general anesthesia immediately before extubation, patients were unaware of group allocation. Postoperative outcomes were assessed by an anesthesiologist who was blinded to the treatment groups.

### General anesthesia and surgical technique

All patients received premedication with 2 mg intravenous midazolam. Standard monitoring, including electrocardiography, non-invasive blood pressure, and pulse oximetry, was applied in the operating room. General anesthesia was induced with intravenous propofol (2–2.5 mg/kg), fentanyl (1–1.5 µg/kg), and rocuronium (0.6 mg/kg). After tracheal intubation, anesthesia was maintained with sevoflurane in a mixture of oxygen and fresh air, along with a remifentanil infusion (50 µg/mL) at a rate of 0.05–2 µg/kg/min. Mechanical ventilation was set with a tidal volume of 6–8 mL/kg, fresh gas flow of 2 L/min, end-tidal CO₂ maintained between 30 and 35 mmHg, and peak airway pressure limited to 30 cmH_2_O.

The same surgical group conducted LC on all patients using the conventional 4-port approach. Port placements included an intraumbilical port (5 mm), a subxiphoid port (15–20 mm), a port at the intersection of the right lateral umbilical line and anterior axillary line (5 mm), and a symmetrical assistant port (5 mm). Ondansetron (4 mg) was administered intravenously for prophylaxis of nausea and vomiting. After adequate spontaneous respiration was observed, patients were extubated and transferred to the Post-Anesthesia Care Unit (PACU).

### Rectointercostal fascial plane block (RIFPB)

After completion of the surgical procedure and following skin closure, bilateral RIFPB was performed in the supine position while the patient remained under general anesthesia and before extubation. A 22-gauge, 80-mm block needle (Braun Stimuplex 360°) and a high-frequency linear ultrasound transducer (11–12 MHz, GE Vivid Q) were utilized. The transducer was initially placed transversely over the sternum, advanced caudally, rotated into the sagittal plane, and positioned 3–4 cm lateral and 3–4 cm caudal to the epigastrium. The needle was positioned using an in-plane approach in a caudal-to-cranial direction after the rectus abdominis muscle and the sixth and seventh costal cartilages were visible (Fig. 1). Hydrodissection was performed with 5 mL of saline at the attachment point of the rectus abdominis muscle to the costal cartilage to confirm the correct fascial plane. After that, each side received an injection of 30 mL of 0.25% bupivacaine, for a total amount of 60 mL. The spread of the local anesthetic was visually confirmed under ultrasound guidance. The block was performed according to the technique described by Tulgar et al. [12].

In the control group, port-site infiltration with 30 mL of 0.25% bupivacaine was performed by the surgical team as part of routine analgesic management. Port-site infiltration was not performed in the RIFPB group in order to evaluate the isolated analgesic effect of the RIFPB technique.

### Postoperative analgesia protocol and pain assessment

Approximately 20 min before skin closure, intravenous ibuprofen 400 mg and tramadol 100 mg were administered. Postoperatively, all patients received routine ibuprofen 400 mg three times daily. Pain intensity was assessed by a blinded anesthetist using the Numeric Rating Scale (NRS) at the 1st, 3rd, 6th, 12th, 18th, and 24th postoperative hours. When NRS scores were ≥ 4, intravenous tramadol 0.5–1 mg/kg was administered as rescue analgesia. The amount of opioids consumed overall, the frequency of nausea and vomiting, itching, allergic reactions, and block-related issues were all noted.

### Outcomes

The requirement for rescue analgesia was the primary outcome. Secondary outcomes were the frequency of side effects, such as nausea, vomiting, and itching, and postoperative pain intensity as measured by the NRS. The requirement for rescue analgesia and the occurrence of adverse effects were recorded as dichotomous variables (yes/no).

### Sample size

For the primary outcome, the requirement for rescue analgesia, two independent proportions (RIFPB vs. Control; two-tailed, α = 0.05) were compared using an initial power calculation in G*Power (version 3.1, Düsseldorf, Germany). Preliminary data suggested event rates of 15% in the RIFPB group and 55% in the Control group (absolute risk difference of 40 percentage points; approximate odds ratio = 6.92). In order to get 90% statistical power to detect the observed effect, a required sample size of n = 64 (32 patients per group) was determined using a z-test for the difference in proportions and a 1:1 allocation. To account for an anticipated 20% attrition rate due to dropouts or missing data, the target sample size was increased to 76 participants.

### Statistical analysis

The distribution of variables was assessed using the Shapiro–Wilk test to evaluate normality. Continuous variables were presented as mean ± standard deviation or median (interquartile range), as appropriate. Normally distributed variables were analyzed using the independent samples t-test, whereas non-normally distributed variables were analyzed using the Mann–Whitney U test. A p value < 0.05 was considered statistically significant. Statistical analyses were performed using SPSS version 25.0 (SPSS Inc., Chicago, IL, USA). A multivariate logistic regression analysis was performed to adjust for potential confounding factors, specifically the ASA physical status, on the primary outcome of rescue analgesia requirement.

## Results

**Fig. 2:**
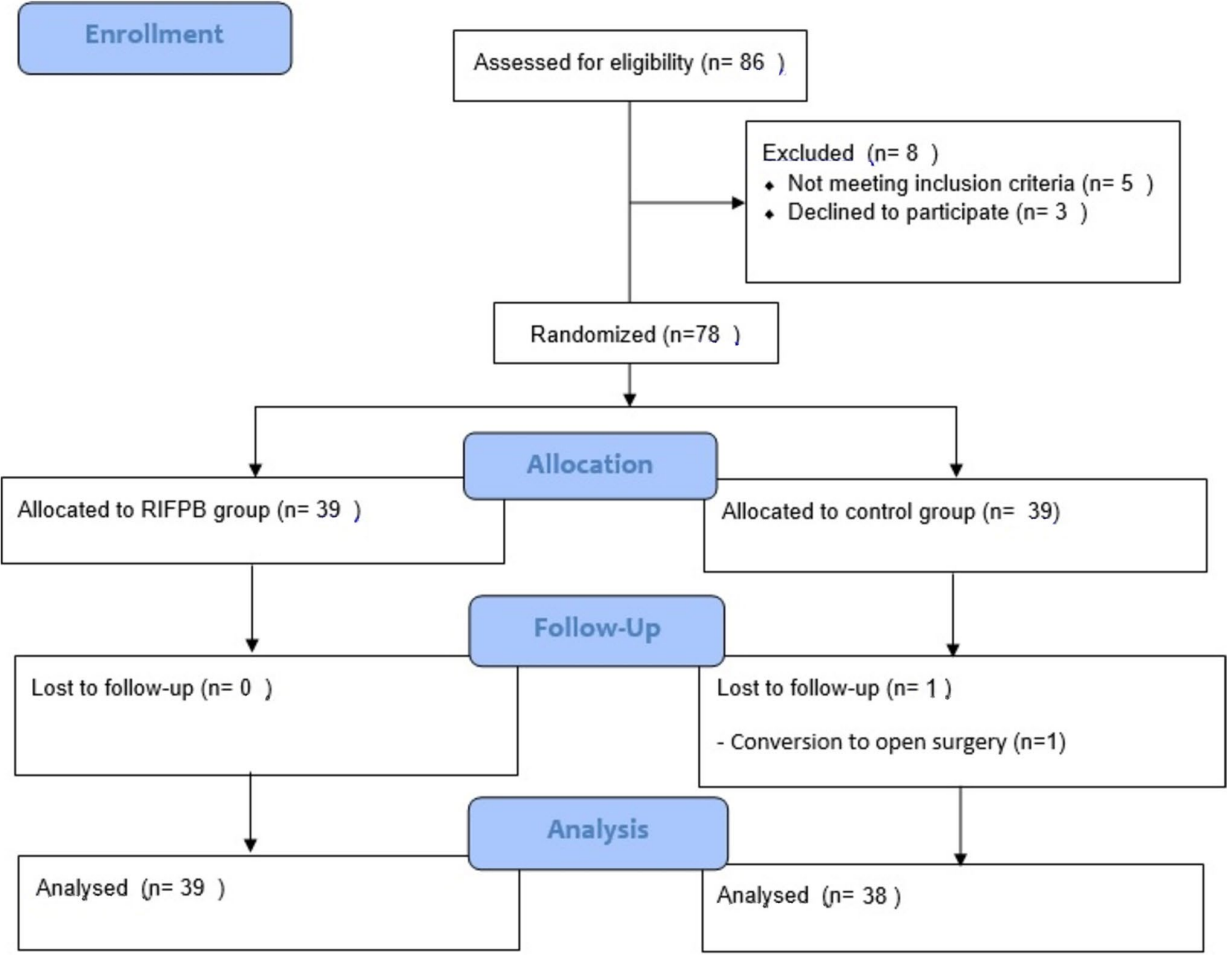
CONSORT flow diagram of the study

During the trial, the CONSORT flow diagram was utilized for participant enrollment (Fig. 2). The trial comprised 77 patients in total, 39 of whom were assigned to the RIFPB group and 38 to the control group. The groups were comparable in terms of demographic characteristics, including age, sex, height, weight, and ASA physical status. In addition, duration of surgery and anesthesia did not differ significantly between the groups (p > 0.05 for all comparisons) (Table 1).

**Table 1:** Comparison of demographic data and duration times of surgery and anesthesia

	RIFPB group n:39	Control group n:38	p
Age	44 [33–52]	40 [32–52]	0.943
Gender (M/F)	24/15	22/16	0.744
ASA (I/II)	21/18	13/25	0.059
Height (cm)	166[163–175]	169 [161–175]	0.787
Weight (kg)	81 [72–93]	82 [75–91]	0.963
Duration of surgery (min)	65 [50–80]	60 [50–76]	0.407
Duration of anesthesia (min)	90 [80–110]	85 [73–106]	0.079

Values are presented as median (interquartile range, 25th–75th percentiles) or number of patients (n)

*ASA* American Society of Anesthesiologists, *m* male, *f* female, *cm* centimeter, *kg* kilogram, *min* minutes

p-values were obtained using the Pearson χ^2^ test for categorical variables and the Mann-Whitney U test for continuous variables

The incidence of rescue analgesia use was significantly lower in the RIFPB group compared with the control group. Rescue analgesia was required in 7 patients (17.9%) in the RIFPB group and 21 patients (55.3%) in the control group (p = 0.001). The RIFPB group consistently received a considerably lower total rescue analgesic dose [0 (0–0) mg] than the control group [40 (0–50) mg] (p = 0.001) (Table 2). The multivariate logistic regression analysis demonstrated that the RIFPB group remained a significant independent predictor of reduced rescue analgesia requirement (OR: 0.081, 95% CI 0.027–0.245, p < 0.001), regardless of the ASA status (p = 0.785).

**Table 2:** The comparison of consumptions and the use of rescue analgesia between groups

	RIFPB group n:39	Control group n:38	p
Rescue analgesia (Y/N)	7/32	21/17	**0.001**
Rescue dose (mg) (median and interquartile range)	0 [0–0]	40 [0–50]	**0.001**
Rescue dose (mg) (mean ± SD)	8.20 ± 17.90	24.73 ± 22.98	0.001*

Values are presented as number of patients (n) or median (interquartile range, 25th–75th percentiles). p values were obtained using the Pearson χ^2^ test for categorical variables and the Mann–Whitney U test for continuous variables

*Y* yes (number of patients who required rescue analgesia), *N* no

Values written in bold indicate statistical significance

*p values were obtained using the Independent Samples t-test

At rest, NRS scores were significantly lower in the RIFPB group at the 1st, 3rd, 6th, and 12th postoperative hours (p = 0.002, p = 0.001, p = 0.015, and p = 0.005, respectively). No statistically significant differences were observed between the groups at the 18th and 24th postoperative hours (p = 0.377 and p = 0.719, respectively). Dynamic NRS scores were also lower in the RIFPB group at the 1st, 3rd, 6th, and 12th postoperative hours (p = 0.009, p = 0.001, p = 0.001, and p = 0.005, respectively). At the 18th and 24th hours, pain scores were comparable between the groups (p = 0.719 and p = 0.476, respectively) (Table 3).

**Table 3:** Comparisons of static and dynamic NRS assessment between groups

	RIFPB group n:39	Control group n:38	p
At rest			
1st hour	0 [0–2]	3 [0–4]	**0.002**
3th hour	0 [0–1]	2 [0–3]	**0.001**
6th hour	0 [0–1]	1 [0–2]	**0.015**
12th hour	0 [0–1]	1 [0–2]	**0.005**
18th hour	0 [0–0]	0 [0–1]	0.377
24th hour	0 [0–0]	0 [0–0]	0.719
On movement			
1st hour	0 [0–1]	2 [0–2]	**0.009**
3th hour	0 [0–0]	1 [0–2]	**0.001**
6th hour	0 [0–0]	0 [0–1]	**0.001**
12th hour	0 [0–0]	1 [0–2]	**0.005**
18th hour	0 [0–0]	0 [0–1]	0.719
24th hour	0 [0–1]	0 [0–0]	0.476

Values are presented as median (interquartile range, 25th–75th percentiles). p values were obtained using the Mann–Whitney U test

*NRS* numeric rating scale

Values written in bold indicate statistical significance

**Fig. 3:**
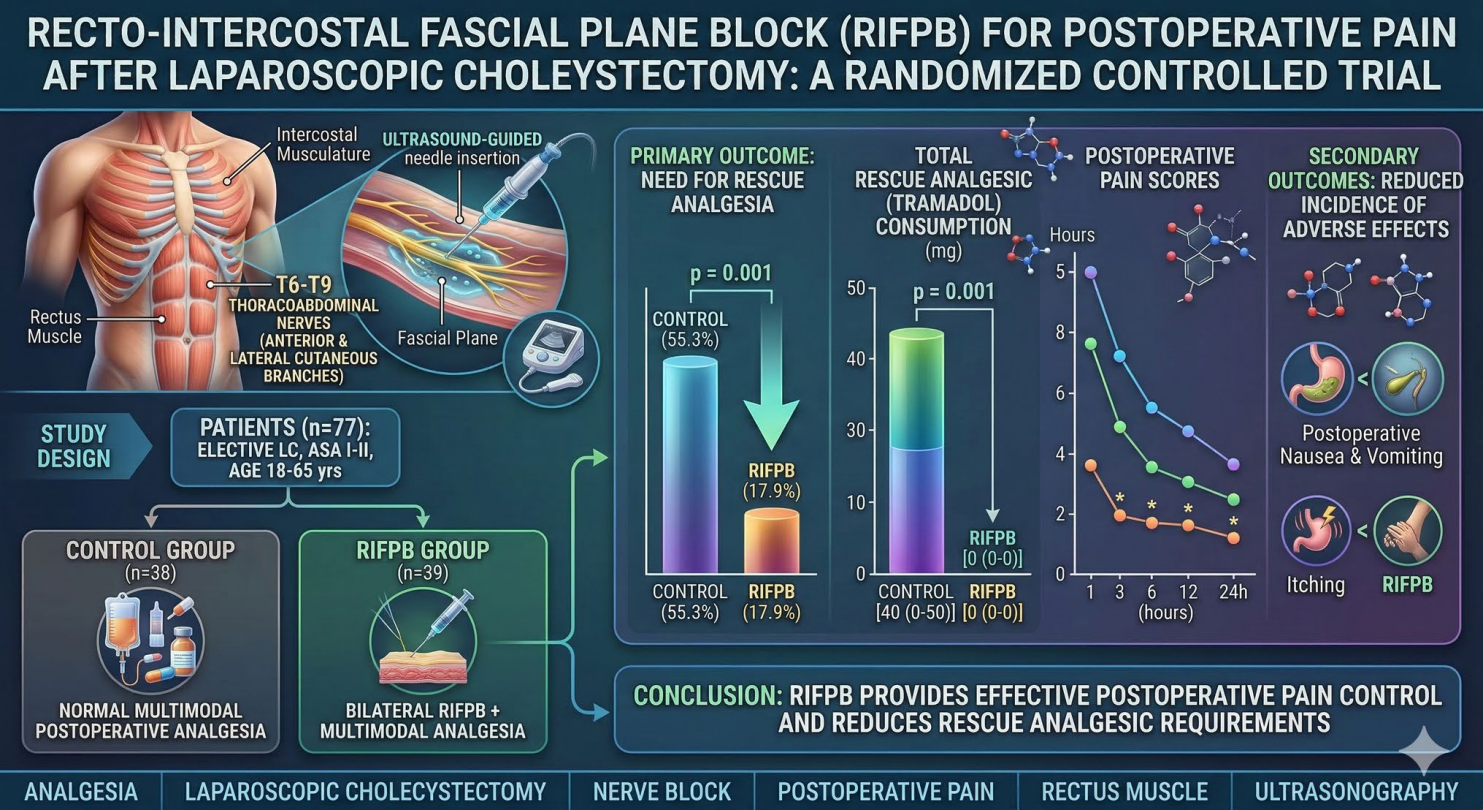
Visual abstract of the study

The RIFPB group experienced fewer postoperative adverse effects, including as nausea and vomiting. Two patients in the RIFPB group and fifteen patients in the control group had nausea (p = 0.0003). One patient in the RIFPB group and eleven patients in the control group experienced vomiting (p = 0.0015). Thirteen patients in the control group and zero in the RIFPB group reported itching (p < 0.0001) (Table 4). No signs or symptoms suggestive of local anesthetic systemic toxicity (LAST) were observed in any patient during the perioperative period. Please see Fig. 3 for visual abstract.

**Table 4:** Comparison of the incidence of side effects between groups

	RIFPB group n:39	Control group n:38	p
Nausea (Y/N)	2/37	15/23 (39.5%)	**0.0003**
Vomiting (Y/N)	1/38	11/27 (28.9%)	**0.0015**
Itching (Y/N)	–	13/25 (34.2%)	** < 0.0001**

Values are presented as number of patients (n). p values were obtained using the Pearson χ^2^ test or Fisher’s exact test, as appropriate

*Y* yes, *N* no

Values written in bold indicate statistical significance

## Discussion

According to our findings, RIFPB provided significantly lower postoperative pain scores and reduced opioid consumption compared with the control group in patients undergoing LC. These results suggest that this newly described block technique may represent a safe and effective analgesic alternative for laparoscopic upper abdominal surgeries. In addition, the lower incidence of opioid-related adverse effects, including itching, nausea, and vomiting, observed in the RIFPB group may be related to the reduced requirement for rescue opioid analgesia. Since tramadol was used as the rescue analgesic in our study, the reduced opioid consumption in the RIFPB group may have contributed to the lower rate of these adverse effects.

The anatomical spread of RIFPB is particularly relevant for targeting incision-related pain in the epigastric and subcostal regions [12]. In addition, application of the block in a fascial plane deep to the rectus abdominis muscle, outside the effective range of pectoral and parasternal blocks, provides potential coverage of both the sub-sternal and epigastric areas. The anatomical location of RIFPB, targeting the plane between the rectus abdominis muscle and the sixth–seventh costal cartilages, encompasses the dominant pain components of LC, including trocar site pain, epigastric incision pain, and diaphragmatic or peritoneal irritation associated with CO₂ insufflation. The spread pattern of RIFPB, which may reach both anterior cutaneous branches and lateral branches, covers the regions where pain is typically perceived after LC and may result in a more homogeneous analgesic distribution in the upper abdomen. Although fascial plane blocks primarily provide somatic analgesia, their effect on visceral pain is not yet fully understood. One possible explanation is that interfascial spread of local anesthetic may extend beyond the intended somatic branches and may indirectly influence visceral afferent or sympathetic pathways. In addition, systemic absorption of local anesthetic may contribute to the overall analgesic effect. These mechanisms remain hypothetical and may partly explain the reduction in postoperative pain observed after RIFPB.

Following its initial description, RIFPB has been applied in various studies involving cardiac surgery, where it was shown to significantly reduce postoperative pain scores and opioid consumption [14–16]. Considering the dermatomal distribution affected by RIFPB, these findings suggest that the block may also be effective in upper abdominal surgeries. Tulgar et al. further demonstrated that local anesthetic administered here spreads widely from the sternum to the midaxillary line, supporting the potential effectiveness of RIFPB [17].

In a randomized study conducted by Shin et al., oblique subcostal TAP block (OSTAP) was reported to be effective for early postoperative pain control after LC [10]. However, a systematic review by Peng et al. emphasized that TAP blocks provide analgesia for only 8–12 h and do not completely eliminate the need for additional analgesics [11]. The more cranial application of RIFPB compared with TAP block and the ability of the local anesthetic to spread to both anterior and lateral branches may allow this technique to overcome the limited duration of analgesia observed with TAP blocks. In the present study, RIFPB was found to be effective in pain management throughout the 24 h postoperative period.

In a randomized study by Güngör et al. comparing M-TAPA block with local infiltration in patients undergoing LC, significantly lower pain scores and opioid consumption were reported in the M-TAPA group [8]. Similarly, Bilge et al. compared M-TAPA with oblique subcostal TAP block and demonstrated that M-TAPA provided a longer duration of analgesia [18]. The more medial and cranial anatomical location of RIFPB compared with M-TAPA may offer a stronger blockade of the epigastric region. Furthermore, visualization of the subcostal margin with ultrasound during M-TAPA block application may be challenging, particularly in obese patients; therefore, blocks performed in more superficial planes may offer practical advantages. In this context, RIFPB may represent a different anatomical target compared with deeper fascial plane blocks and may provide an alternative regional analgesic approach [19].

Ciftci et al. compared M-TAPA and EOIPB blocks and reported that both techniques significantly improved postoperative analgesia; however, the M-TAPA group exhibited lower pain scores and reduced tramadol consumption within the first 12 h [20]. This study demonstrated that M-TAPA effectively blocks the T6–T10 dermatomes via the perichondral course of thoracoabdominal nerves, whereas EOIPB provides a more superficial spread through lateral branches. Similarly, comparisons between unilateral EOIPB and subcostal TAP block revealed superior early analgesic effects with EOIPB [21]. Additionally, Korkusuz et al. reported significantly lower pain scores and reduced tramadol consumption in patients receiving bilateral EOIPB compared with a control group undergoing LC [9]. Although EOIPB blocks the T6–T10 segments laterally, its contribution to visceral pain control is limited. In contrast, RIFPB targets the same dermatomal area via an anterior approach, inhibiting conduction through both medial and lateral branches. The anteromedial spread of RIFPB may provide a clinical advantage, particularly for epigastric incision pain. Compared with the lateral approach of M-TAPA, RIFPB may offer more selective and effective analgesia in the epigastric region, while providing broader dermatomal coverage than EOIPB. Therefore, RIFPB may be more effective in managing the mixed pain components, including visceral pain, associated with LC. The anteromedial location of RIFPB allows more targeted analgesia, particularly for epigastric and subcostal pain, and may contribute to a reduction in opioid-related adverse effects such as nausea and vomiting.

Khalil et al. compared erector spinae plane (ESP) block with oblique subcostal TAP block and reported no difference in terms of analgesic efficacy between the two techniques, although ESP block required a longer application time [22]. While ESP block provides wide dermatomal coverage at the thoracic level, its ability to target the epigastric region, where pain is most prominent after LC, is limited. In contrast, the direct anatomical targeting of this region by RIFPB may explain its more selective and potent analgesic effect. Gangadhar et al. reported that a combination of EOIPB and rectus sheath block resulted in lower pain scores and reduced opioid requirements compared with trocar site infiltration anesthesia [23]. This finding highlights the clinical advantage of combining different fascial plane blocks. Similarly, RIFPB may be combined with parasternal or rectus sheath blocks to expand both medial and lateral sensory coverage in laparoscopic surgeries, further supporting the consistency of our findings with the existing literature and their clinical applicability.

This study has several limitations. First, the sensory dermatomal distribution following RIFPB was not evaluated using objective assessment methods, which limits precise identification of the dermatomes responsible for the observed analgesic effect. Second, the relatively small sample size may limit the generalizability of the results, and confirmation in larger studies is warranted. Third, a relatively large volume of local anesthetic was used for RIFPB (30 mL per side; total 60 mL), corresponding to a total dose of 150 mg of 0.25% bupivacaine. This volume was higher than the amount used for port-site infiltration in the control group (30 mL), which may have contributed to the observed analgesic differences between the groups. Although this dose approaches the upper recommended range depending on patient weight, no signs of LAST were observed. Future studies may evaluate whether similar analgesic efficacy can be achieved with lower volumes of local anesthetic. In addition, although baseline characteristics were generally comparable between groups, a slight imbalance in ASA physical status distribution was observed. Although this difference was not statistically significant, it may represent a potential confounding factor and should be interpreted with caution. Furthermore, this was a single-center study, which may limit the generalizability of the findings. Finally, long-term postoperative outcomes, such as chronic pain or patient satisfaction beyond the first 24 h, were not evaluated. Considering these limitations, future prospective studies with larger sample sizes and objective dermatomal assessments are needed to define the optimal application parameters of this technique.

## Conclusion

The results of this study suggest that RIFPB may be a safe and effective technique that can be integrated into multimodal analgesia protocols following LC. However, multicenter, large-scale, randomized controlled studies evaluating different local anesthetic concentrations, unilateral or bilateral applications, and combination strategies with other blocks are required to strengthen the generalizability of these findings.

## Data Availability

The datasets generated during and/or analyzed during the current study are available from the corresponding author on reasonable request.
